# A Importância da Avaliação Eletrovetorcardiográfica dos Bloqueios Intraventriculares e sua Relação com o Ciclo Cardíaco

**DOI:** 10.36660/abc.20240767

**Published:** 2025-02-21

**Affiliations:** 1 Universidade de São Paulo Hospital das Clínicas FMUSP Faculdade de Medicina São Paulo SP Brasil Unidade Clínica de Eletrocardiografia – Instituto do Coração (InCor) – Hospital das Clínicas FMUSP – Faculdade de Medicina, Universidade de São Paulo, São Paulo, SP – Brasil

**Keywords:** Vetorcardiografia, Bloqueio Cardíaco, Eletrocardiografia

O impacto dos distúrbios de condução intraventricular na ativação miocárdica e na função ventricular tem sido debatido há muito tempo. No entanto, com o advento da terapia de ressincronização cardíaca, esse tópico ganhou ainda mais atenção nas últimas duas décadas.^1,2^

Naquela época, nossa equipe utilizou mapeamento eletrocardiográfico de superfície e mapas de linhas isócronas para avaliar o tempo médio de ativação em três regiões específicas do coração: o ventrículo direito (VD), o ventrículo esquerdo (VE) e a região anterosseptal. Essa avaliação foi conduzida em pacientes com fração de ejeção do VE menor que 40%, exibindo bloqueio do ramo esquerdo (BRE) e com duração média do QRS de 180 ms.^1,2^ A ativação elétrica cardíaca foi avaliada em três grupos de pacientes: 1) pacientes com BRE nativo, 2) pacientes sob estimulação VD e 3) pacientes sob estimulação biventricular (EBV). Em indivíduos com BRE, os tempos médios de ativação do VD e da ativação anterosseptal foram atrasados em comparação ao VE, levando a uma perda de sincronia. Da mesma forma, o tempo de ativação do grupo de estimulação VD estendeu-se além do normal, com uma diferença mais significativa entre região septal e VE. Os tempos de ativação do VE e do VD foram semelhantes durante a EBV, e a ativação septal se aproximou das condições normais e nativas do BRE. Esses achados sugeriram que a ativação elétrica prolongada do VD pela EBV, que às vezes pode passar despercebida na presença de BRE, pode ser um critério relevante na seleção de candidatos para terapia de ressincronização cardíaca.^1,2^

A eletrocardiografia de superfície é atualmente usada para classificar os diferentes tipos de bloqueios de condução intraventricular. Ao mesmo tempo, a ecocardiografia, com suas inúmeras técnicas, permite a análise dos efeitos funcionais dessas anormalidades elétricas.^3^ Recentemente, a aplicação de transformações matemáticas aos sinais de eletrocardiograma de superfície simplificou a aquisição de vetorcardiogramas.^4^ Este método aumenta a precisão do eletrocardiograma na identificação de atrasos na ativação elétrica e na repolarização cardíaca, o que permite uma análise vetorial simultânea em três planos e uma decomposição detalhada dos loops elétricos das ondas P e T, e dos complexos QRS, divididos em fases inicial e terminal, identificando os pontos de maior amplitude.^5^

A análise dos efeitos funcionais do bloqueio completo do ramo esquerdo no ciclo cardíaco demonstrou um prolongamento da sístole e encurtamento da diástole, entre outros impactos negativos na dinâmica do VE resultantes dessa perturbação elétrica.^6^

Muitos outros estudos investigaram o impacto do BRE na função cardíaca e na resposta à terapia de ressincronização cardíaca. As implicações de outros distúrbios de condução intraventricular na motilidade cardíaca ainda não foram caracterizadas. Na edição atual deste periódico, os autores do manuscrito intitulado "Impacto dos Bloqueios Intraventriculares na Dinâmica do Ciclo Cardíaco: Uma Análise ECO e Vetorcardiográfica"^7^ estudaram como os bloqueios intraventriculares (BIV) podem causar atrasos e alterar o ciclo ventricular. Neste estudo transversal, os autores avaliaram 328 indivíduos consecutivos sem doença cardíaca estrutural apresentando eletrocardiograma normal ou diferentes tipos de BIV. Todos os pacientes foram submetidos a uma análise abrangente com ecocardiogramas transtorácicos e vetorcardiogramas (VCG) simultâneos para investigar os aspectos eletromecânicos do ciclo cardíaco. Os traçados do vetorcardiograma fornecem informações valiosas, ilustrando as alterações sequenciais no sistema de condução elétrica do coração. Os autores puderam identificar distúrbios de condução e suas associações avaliando os loops de P, QRS e T, descrevendo informações detalhadas sobre atrasos de condução em todas as fases de ativação elétrica do coração. Os autores demonstraram em seu estudo que BIV, além do BRE, podem alterar o ciclo cardíaco, com ênfase especial na associação do bloqueio do ramo direito (BRD) com um bloqueio fascicular antero-superior esquerdo (BDASE). Pacientes que apresentam BRD e BDASE podem se beneficiar da terapia de ressincronização cardíaca; no entanto, mais estudos são necessários para avaliar tal terapia em grandes populações.
