# Supplementary material for: A Importância da Avaliação Eletrovetorcardiográfica dos Bloqueios Intraventriculares e sua Relação com o Ciclo Cardíaco
Source: Arq Bras Cardiol. 2025 Feb 21;121(12):e20240767. [Article in Portuguese] doi: 10.36660/abc.20240767 (PMC11884667; doi:10.36660/abc.20240767)
Supplement: Translation

# Translation: The Importance of Electrovectorcardiographic Assessment of Intraventricular Blocks and their Relationship with the Cardiac Cycle

**DOI:** 10.36660/abc.20240767i

**Affiliations:** 1 São Paulo SP Brazil Unidade Clínica de Eletrocardiografia – Instituto do Coração (InCor) - Hospital das Clínicas FMUSP – Faculdade de Medicina, Universidade de São Paulo, São Paulo, SP – Brazil

**Keywords:** Vectorcardiography, Heart Block, Electrocardiography

The impact of intraventricular conduction disorders on myocardial activation and ventricular function has long been debated. However, with the advent of cardiac resynchronization therapy, this topic has gained even more attention during the last two decades.^1,2^

At that time, our team utilized surface electrocardiographic mapping and isochronal line maps to assess the mean activation time in three specific regions of the heart: the right ventricle (RV), the left ventricle (LV), and the anteroseptal region. This evaluation was conducted on patients with a left ventricular ejection fraction of less than 40%, who exhibited left bundle branch block (LBBB), and had a mean QRS duration of 180 ms.^1,2^ The cardiac electrical activation was evaluated in three groups of patients: 1) patients with native LBBB, 2) patients under RV pacing, and 3) patients under biventricular pacing (BVP). In individuals with LBBB, the RV and anteroseptal mean activation time were delayed compared to the LV, leading to a loss of synchrony. Similarly, the RV pacing group's activation time extended beyond normal, with a more significant difference between the septal area and LV. LV and RV activation times were similar during BVP, and the septal activation approached the normal and native LBBB conditions. Those findings suggested that the prolonged RV electrical activation by BVP, which sometimes may go unnoticed in the presence of LBBB, could be a relevant criterion in selecting candidates for cardiac resynchronization therapy.^1,2^

Surface electrocardiography is currently used to classify the different types of intraventricular conduction blocks. At the same time, echocardiography, with its numerous techniques, enables the analysis of the functional effects of these electrical abnormalities.^3^ Recently, applying mathematical transformations to surface electrocardiogram signals has simplified the acquisition of vectorcardiograms (VCG).^4^ This method enhances the electrocardiogram accuracy in identifying delays in electrical activation and cardiac repolarization, which allows a simultaneous vector analysis in three planes and a detailed decomposition of the electrical loops of the P and T waves, and the QRS complexes, divided into initial and terminal phases, pinpointing the points of highest amplitude.^5^

The analysis of the functional effects of complete LBBB on the cardiac cycle has demonstrated a prolongation of systole and shortening of diastole, among other negative impacts on LV dynamics resulting from this electrical disturbance.^6^

Many other studies investigated the impact of LBBB on cardiac function and response to cardiac resynchronization therapy. The implications of other intraventricular conduction disorders on heart motility have not yet been characterized. In this journal's current issue, the authors of the manuscript entitled "Impact of Intraventricular Blocks on Cardiac Cycle Dynamics: An Echocardiographic and Vectorcardiographic Analysis"^7^ studied how intraventricular blocks (IVB) may cause delays and alter the ventricular cycle. In this cross-sectional study, the authors evaluated 328 consecutive individuals without structural heart disease presenting with either normal electrocardiogram or different types of IVB. All patients underwent a comprehensive analysis with simultaneous transthoracic echocardiograms and VCG to investigate the electromechanical aspects of the cardiac cycle. VCG traces provide valuable information, illustrating the sequential alterations in the heart's electrical conduction system. The authors could identify conduction disturbances and their associations by evaluating the loops of P, QRS, and T, describing detailed information about conduction delays at all the heart's electrical activation phases. The authors demonstrated in their study that IVB, other than LBBB, might alter the cardiac cycle, with special emphasis on the association of the right bundle branch block (RBBB) with a left anterior fascicular block (LAFB). Patients presenting RBBB and LAFB may benefit from cardiac resynchronization therapy; however, more studies are needed to evaluate such therapy in large populations.
